# Supplementary material for: Lottery before peer review is associated with increased female representation and reduced estimated economic cost in a German funding line
Source: Nat Commun. 2025 Nov 6;16:9824. doi: 10.1038/s41467-025-65660-9 (PMC12592401; doi:10.1038/s41467-025-65660-9)
Supplement: Supplementary file 1 — Supplementary Information [file 41467_2025_65660_MOESM1_ESM.pdf]

Table S1. Demographics of applicants in survey 1.

		n	valid %
Age			
	20-25	4	(0.5)
	26-30	74	(8.8)
	31-35	117	(14.0)
	36-40	151	(18.0)
	41-45	189	(22.6)
	46-50	115	(13.7)
	51-55	113	(13.5)
	56-60	55	(6.6)
	61-65	19	(2.3)
	Missing	57	
Gender			
	Female	443	(54.2)
	Male	367	(44.9)
	Diverse	7	(0.9)
	Missing	77	
Status group			
	PhD Student	123	(14.6)
	PostDoc	108	(12.8)
	Lecturer	107	(12.7)
	Professor (assistant)	21	(2.5)
	Professor (associated)	235	(27.8)
	Professor (chair)	68	(8.0)
	Research management	109	(12.9)
	Other	74	(8.8)
	Missing	49	

Note.

Table S2. Demographics of applicants in survey 2.

		n	valid %
Age			
	20-25	0	(0)
	26-30	12	(16.9)
	31-35	8	(11.3)
	36-40	8	(11.3)
	41-45	18	(25.4)
	46-50	5	(7.0)
	51-55	6	(8.5)
	56-60	9	(12.7)
	61-65	5	(7.0)
	Missing	10	
Gender			
	Female	34	(48.6)
	Male	36	(51.4)
	Diverse	0	(0)
	Missing	11	
Status group			
	PhD Student	14	(19.7)
	PostDoc	9	(12.7)
	Lecturer	4	(5.6)
	Professor (assistant)	0	(0)
	Professor (associated)	22	(31.0)
	Professor (chair)	11	(15.5)
	Research management	7	(9.9)
	Other	4	(5.6)
	Missing	10	

Note.

Table S3. Demographics of reviewers in survey 3.

	n	valid %
Status group		
Student	33	(27.0)
PostDoc/Lecturer	20	(16.4)
Professor	38	(31.1)
Management/Didactics	31	(25.4)
Missing	6	

Note.

Table S4. Descriptive statistics for satisfaction and preferences.

Variable	Mean	SD	Median	Skew	Kurtosis
Applicants					
Satisfaction	65.19	26.61	69	-0.62	-0.26
Preference					
Overall	50.30	31.23	50	-0.11	-1.13
Amount of work	77.64	29.56	88	-1.61	1.46
Own chance of success	40.50	33.25	36	0.43	-1.06
Fairness	40.62	35.97	34	0.41	-1.28
Quality	34.44	31.09	28	0.63	-0.73
Reviewers					
Satisfaction	72.21	21.59	79	-0.81	-0.18
Preference					
Overall	50.01	37.81	58	-0.03	-1.65

Note.

Table S5. Requested funding volume of applications.

Funding year		Mean	Std	Median	Min	Max
2022	submitted	212,770.15 €	143,909.87 €	178,149.20 €	5,268.00 €	625,000.00 €
	selected	196,305.34 €	123,205.56 €	175,300.00 €	11,500.00 €	625,000.00 €
2023	submitted	290,242.64 €	97,093.10 €	290,242.64 €	25,528.71 €	400,000.00 €
	selected	287,505.10 €	100,126.86 €	287,505.10 €	25,528.71 €	400,000.00 €
2024	submitted	332,030.52 €	81,535.88 €	332,030.52 €	32,032.29 €	399,999.60 €
	selected	338,081.61 €	78,182.61 €	338,081.61 €	49,701.35 €	399,997.73 €

Note.
