# Supplementary material for: Lottery before peer review is associated with increased female representation and reduced estimated economic cost in a German funding line
Source: Nat Commun. 2025 Nov 6;16:9824. doi: 10.1038/s41467-025-65660-9 (PMC12592401; doi:10.1038/s41467-025-65660-9)
Supplement: Supplementary file 2 — Reporting Summary [file 41467_2025_65660_MOESM2_ESM.pdf]

nature portfolio

Corresponding author(s): Sören Krach

Last updated by author(s): 18 August 2025

Reporting Summary

Nature Portfolio wishes to improve the reproducibility of the work that we publish. This form provides structure for consistency and transparency in reporting. For further information on Nature Portfolio policies, see our Editorial Policies and the Editorial Policy Checklist.

Statistics

For all statistical analyses, confirm that the following items are present in the figure legend, table legend, main text, or Methods section.

n/a	Confirmed	
☐	☒	The exact sample size (n) for each experimental group/condition, given as a discrete number and unit of measurement
☐	☒	A statement on whether measurements were taken from distinct samples or whether the same sample was measured repeatedly
☐	☒	The statistical test(s) used AND whether they are one- or two-sided<br>*Only common tests should be described solely by name; describe more complex techniques in the Methods section.*
☒	☐	A description of all covariates tested
☐	☒	A description of any assumptions or corrections, such as tests of normality and adjustment for multiple comparisons
☐	☒	A full description of the statistical parameters including central tendency (e.g. means) or other basic estimates (e.g. regression coefficient) AND variation (e.g. standard deviation) or associated estimates of uncertainty (e.g. confidence intervals)
☐	☒	For null hypothesis testing, the test statistic (e.g. F, t, r) with confidence intervals, effect sizes, degrees of freedom and P value noted<br>*Give P values as exact values whenever suitable.*
☒	☐	For Bayesian analysis, information on the choice of priors and Markov chain Monte Carlo settings
☒	☐	For hierarchical and complex designs, identification of the appropriate level for tests and full reporting of outcomes
☒	☐	Estimates of effect sizes (e.g. Cohen's d, Pearson's r), indicating how they were calculated

Our web collection on statistics for biologists contains articles on many of the points above.

Software and code

Policy information about availability of computer code

Data collection	SoSci Survey (Version 3.2.31) was used to collect all survey data. Data of applicants' gender was collected by the Stiftung Innovation in der Hochschullehre which administers the funding line; see https://doi.org/10.17605/OSF.IO/4UFRB.
Data analysis	R version 4.3.0 (2023-04-21 ucrt) was used to analyse the data<br>Dependencies:<br>attached base packages:<br><br>[1] stats graphics grDevices utils datasets methods base<br><br>other attached packages:<br><br>[1] here_1.0.1 scales_1.3.0 readxl_1.4.2 tibble_3.2.1 purrr_1.0.1 patchwork_1.3.0<br><br>[7] tidyr_1.3.0 dplyr_1.1.2 ggforce_0.4.2 ggplot2_3.5.1<br><br>loaded via a namespace (and not attached):<br><br>[1] vctrs_0.6.3 cli_3.6.1 rlang_1.1.4 renv_0.17.3 generics_0.1.3

```
[6] glue_1.6.2      colorspace_2.1-0 rprojroot_2.0.3  fansi_1.0.4     cellranger_1.1.0

[11] tweenr_2.0.3    grid_4.3.0       munsell_0.5.0    MASS_7.3-58.4    lifecycle_1.0.3

[16] compiler_4.3.0  see_0.8.4        polyclip_1.10-6  Rcpp_1.0.10      pkgconfig_2.0.3

[21] rstudioapi_0.14  farver_2.1.1     R6_2.5.1         tidyselect_1.2.0 utf8_1.2.3

[26] pillar_1.9.0    magrittr_2.0.3   tools_4.3.0      withr_2.5.0      gtable_0.3.3
```

For manuscripts utilizing custom algorithms or software that are central to the research but not yet described in published literature, software must be made available to editors and reviewers. We strongly encourage code deposition in a community repository (e.g. GitHub). See the Nature Portfolio guidelines for submitting code & software for further information.

Data

Policy information about availability of data

All manuscripts must include a data availability statement. This statement should provide the following information, where applicable:

- Accession codes, unique identifiers, or web links for publicly available datasets
- A description of any restrictions on data availability
- For clinical datasets or third party data, please ensure that the statement adheres to our policy

All data underlying the figures and all data used to reproduce the analyses are provided. Source data is provided with the figures.

Research involving human participants, their data, or biological material

Policy information about studies with human participants or human data. See also policy information about sex, gender (identity/presentation), and sexual orientation and race, ethnicity and racism.

Reporting on sex and gender

We would like to point out that we put great emphasis on delineating the topic of sex/gender in the present study. In our title, we refer to “gender bias” including both genders and other gender identities where available. In our manuscript, we write the following regarding sex/gender:

“For every dataset, the applicants’ gender was coded based on the available data of the formal assessment during the application process. Each applicant’s gender was classified based on the German salutation that applicants had selected in the respective forms of the submission process: ‘Herr’ [‘Mr’], ‘Frau’ [‘Ms’], and ‘keine’ [‘not specified’]. In case no salutation was specified, gender was classified based on gender self-assessments within the application forms in case they were available (i.e., ‘weiblich’ [‘female’], ‘männlich’ [‘male’], ‘divers’ [‘diverse’], ‘keine Angabe’ [‘not specified’], ‘---’). Based on the available data, ‘Ms’ or ‘female’ was classified as ‘female’, ‘Mr’ or ‘male’ was classified as ‘male’, and all other categories were classified as ‘diverse / not specified’. This classification was reliable, as there was no conflicting gender classifications based on the different self-report sources (salutation or ‘gender’). While we acknowledge that the chosen approach to define gender may be overly simplistic, the present analyses are necessarily limited to the available historical data. Thus, we also restricted our statistical tests to the comparison between male and female applicants and ran one-sided proportion tests, comparing the initial application phase and the funded application within each year the lottery was implemented against the year without lottery (2022).”

In our section in “Biases in research funding” we provide detailed data an sex/gender comparisons.

Reporting on race, ethnicity, or other socially relevant groupings

Except gender, we did not use any other categorization variables in our main manuscript. In the supplement, we report on age, gender, and status group of applicants as self-reported in our two surveys, and on status group of reviewers in the reviewer survey. We decided to collect age in bins of five years for purposes of anonymity, and used reporting options of gender and status group that were as close as possible to demographical data that the foundation collects themselves.

Population characteristics

See above

Recruitment

Invitations to the surveys were sent via e-mail by the foundation to all eligible applicants after the submission of (i) the expression of interest, (ii) the full proposal, and to the reviewers after the collection of reviews. Participants needed to give informed consent and were assured that the participation was anonymous, that the foundation had no access to the survey data, and that their responses would not influence their chances to advance to the next round in any way.
However, since participation was voluntary and not a mandatory part of the application process, the results might be biased in a way that only applicants/reviewers participated who had a quite strong motivation to do so, either due to positive or negative attitudes about the grant distribution approach. We discuss this limitation in the manuscript.

Ethics oversight

All surveys were conducted under block ethics approval to the MPI Decision Lab (RM) granted by the Ethics Council of the Max Planck Society

Note that full information on the approval of the study protocol must also be provided in the manuscript.

Field-specific reporting

Please select the one below that is the best fit for your research. If you are not sure, read the appropriate sections before making your selection.

☐ Life sciences ☒ Behavioural & social sciences ☐ Ecological, evolutionary & environmental sciences

For a reference copy of the document with all sections, see nature.com/documents/nr-reporting-summary-flat.pdf

Behavioural & social sciences study design

All studies must disclose on these points even when the disclosure is negative.

Study description	We show quantitative empirical data about costs, gender equality, and acceptance of a lottery-first approach to grant distribution, i.e. an approach where a lottery determines who is able to submit a full application. We use gender data obtained by the foundation responsible for the grant distribution, as well as survey data which we collected during the most recent installment.
Research sample	All applicants of the Freiraum funding line of the foundation "Stiftung Innovation in der Hochschullehre" were targeted for participation, i.e., personnel from all disciplines within German higher education, including science management. In the first survey, 54.2% of participants were female, 44.9% male and 0.7% identified as diverse. The most frequent age category was 41-45. In the second survey, these percentages were 48.6, 51.4, and 0 with the most frequent age category being 41-45 as well.<br>Additionally, we targeted all people who reviewed the proposals, which additionally included students.<br><br>The sample might not be representative: First, more women replied to our initial survey than were part of the respective stage of the application procedure (45.1% vs 54.2 in the survey). Second, we did not target all researchers, but only those who were involved in German higher education (including management) and applied to the Freiraum funding line. From those, only a subset replied to our survey which might have further influenced representativeness.
Sampling strategy	All applicants of the Freiraum funding line got an invitation to participate in the survey. We did not perform a sample size calculation as it was not possible for us to influence the sample size. For that reason, we restrict the analyses mostly to explorative descriptive statistics.
Data collection	Data collection took part online using SoSci Survey (Version 3.2.31) at time, place and device of participants' convenience. There were no conditions to be blinded, and researchers were not blinded for the hypothesis of the only hypothesis test, i.e. the gender distribution across founding approaches across years.
Timing	First survey: 12 February to 6 March 2024<br>Second survey: 23 May to 30 June 2024<br>Reviewer survey: 24 July to 31 August 2024
Data exclusions	Due to not having activated JavaScript in their browser which was necessary for the correct display of survey items, the data sets of seven applicants in the first survey and one participant in the reviewer survey could not be included in the analyses. In addition, applicants with excessively fast relative response times were excluded from the analyses, as these reflect invalid response behaviour, in accordance with the SoSci survey recommendations (Leiner, 2019). This led to the exclusion of 38 applicants in the first survey and one applicant in the second survey.
Non-participation	The response rate of the first survey was 15.6%, second survey 17.8, and reviewer survey 56.3.
Randomization	There were no experimental groups and no randomization.

Reporting for specific materials, systems and methods

We require information from authors about some types of materials, experimental systems and methods used in many studies. Here, indicate whether each material, system or method listed is relevant to your study. If you are not sure if a list item applies to your research, read the appropriate section before selecting a response.

Materials & experimental systems

n/a	Involved in the study	
☒	☐	Antibodies
☒	☐	Eukaryotic cell lines
☒	☐	Palaeontology and archaeology
☒	☐	Animals and other organisms
☒	☐	Clinical data
☒	☐	Dual use research of concern
☒	☐	Plants

Methods

n/a	Involved in the study	
☒	☐	ChIP-seq
☒	☐	Flow cytometry
☒	☐	MRI-based neuroimaging

Plants

Seed stocks	*Report on the source of all seed stocks or other plant material used. If applicable, state the seed stock centre and catalogue number. If plant specimens were collected from the field, describe the collection location, date and sampling procedures.*
Novel plant genotypes	*Describe the methods by which all novel plant genotypes were produced. This includes those generated by transgenic approaches, gene editing, chemical/radiation-based mutagenesis and hybridization. For transgenic lines, describe the transformation method, the number of independent lines analyzed and the generation upon which experiments were performed. For gene-edited lines, describe the editor used, the endogenous sequence targeted for editing, the targeting guide RNA sequence (if applicable) and how the editor was applied.*
Authentication	*Describe any authentication procedures for each seed stock used or novel genotype generated. Describe any experiments used to assess the effect of a mutation and, where applicable, how potential secondary effects (e.g. second site T-DNA insertions, mosiacism, off-target gene editing) were examined.*
